# RFamide Peptides in Early Vertebrate Development

**DOI:** 10.3389/fendo.2014.00203

**Published:** 2014-12-04

**Authors:** Guro Katrine Sandvik, Kjetil Hodne, Trude Marie Haug, Kataaki Okubo, Finn-Arne Weltzien

**Affiliations:** ^1^Department of Basic Sciences and Aquatic medicine, Norwegian University of Life Sciences, Oslo, Norway; ^2^Institute for Experimental Medical Research, Oslo University Hospital, Oslo, Norway; ^3^Department of Biosciences, University of Oslo, Oslo, Norway; ^4^Department of Aquatic Bioscience, Graduate School of Agricultural and Life Sciences, The University of Tokyo, Bunkyo, Japan

**Keywords:** RFa, NPFF, PrRP, Kiss, GnIH, 26RFa/QRFP, early development, brain development

## Abstract

RFamides (RFa) are neuropeptides involved in many different physiological processes in vertebrates, such as reproductive behavior, pubertal activation of the reproductive endocrine axis, control of feeding behavior, and pain modulation. As research has focused mostly on their role in adult vertebrates, the possible roles of these peptides during development are poorly understood. However, the few studies that exist show that RFa are expressed early in development in different vertebrate classes, perhaps mostly associated with the central nervous system. Interestingly, the related peptide family of FMRFa has been shown to be important for brain development in invertebrates. In a teleost, the Japanese medaka, knockdown of genes in the Kiss system indicates that Kiss ligands and receptors are vital for brain development, but few other functional studies exist. Here, we review the literature of RFa in early vertebrate development, including the possible functional roles these peptides may play.

## Introduction

Neuropeptides with an arginine (R) and an amidated phenylalanine (F)-motif at its C-end (called RFamides or RFa) were first described in mollusks in the 70s [FMRFamide (FMRFa)] (1). Soon after, an RFa was also found in a vertebrate (2). Since then, many RFa have been identified in different vertebrate species, with the most recent group of vertebrate RFa found as late as 2002 (3). Vertebrate RFa are currently divided into five groups: (i) neuropeptide FF (NPFF) group or PQRFa group, consisting of NPFF, neuropeptide AF (NPAF), and neuropeptide SF (NPSF); (ii) prolactin-releasing peptide (PrRP) group, consisting of PrRP20 and PrRP31, crucian carp RFamide (C-RFa), and salmon RFa; (iii) gonadotropin-inhibitory hormone (GnIH) group, including mammalian RFa-related peptides (RFRP-1 and RFRP-3), frog growth hormone-releasing peptide (fGRP), and goldfish LPXRFamide peptide; (iv) kisspeptin group also known as metastin; and finally (v) 26RFa/QRFP, including the peptides 26RFa and 43RFa (QRFP) (4, 5). These peptides have been studied in several different physiological contexts, and are found to have a role in a wide range of processes in vertebrates, as in reproductive behavior and in control of the reproductive axis (6), in pain modulation (7), and in control of feeding (8). However, much more research is needed to fully comprehend the function of RFa in different processes.

Interestingly, FMRFa are expressed in the nervous system at very early developmental stages in several phyla of metazoans, as mollusks (cephalopods and gastropods) (9, 10), cnidarians (11), and annelids (a polychete) (12). This may indicate an evolutionary ancient role of FMRFa in the development of the nervous system. It has also been shown that regeneration of flatworm anterior body fragments are stimulated by RFa (13), further supporting a role for RFa in development of tissues in lower metazoans. Also in vertebrates, the developmental studies of RFa show exciting results. Common for all the peptides is that they seem to be expressed at a very early stage in most vertebrate species studied, ranging from fishes to mammals. It seems that RFa could have important roles in development not yet discovered. This review aims to sum up what is known about the temporal and spatial expression pattern, as well as potential functional roles of the different RFa in vertebrate development, using a comparative approach.

## NPFF Group

The first RFa to be identified in vertebrates was NPFF (also known as F8F-amide) and NPAF (2). Also belonging to this group is the peptide NPSF, and all three peptides are transcribed from the same gene in mammals (5). Genes encoding for NPFF have been identified in many different vertebrate classes, from hagfish to mammals, and members of this group bind to the receptor NPFFR2 (also called GPR74, NPFF2) (5).

Few studies have investigated the expression, location, or function of NPFF during development. However, one study in teleosts (14), one in amphibians (15), one in birds (16), and two studies in mammals (17, 18) show that this peptide is expressed early in embryonic life in different vertebrate classes. In addition, many studies have used polyclonal antibodies against FMRFa that also seem to label the NPFF peptide (see later, and Table 1), providing additional information regarding NPFF expression patterns. Below, a brief description of where NPFF is expressed in adult vertebrates is included, followed by a description of the studies performed during development.

**Table 1:** **Overview of studies of NPFF in vertebrate development**.

RFa and/or receptors	Species	Method	Antibody (or radioligand)	Embryonic stages	Location of peptide/mRNA in early developing central nervous system	Putative functions in early development	Reference
NPFF	Zebrafish (*Danio rerio*)	ISH	–	24, 30, 36 hpf, 2, 3, 4, 7 dpf, adult	Exclusively in large cells of the developing terminal nerve	–	(14)

FMRFa (NPFF + ?)	Zebrafish and sterlet (*Acipenser ruthenus*)	ir	Pol 1:1000-1:20000 rabbit anti-FMRFa (Phoenix/Incstar)	24–60 hpf and 5 dpf zebrafish, juvenile sterling	Developing terminal nerve, hyp	Involvement in brain functions	(19)

FMRFa (NPFF + ?)	Brown trout (*Salmo trutta fario*)	ir	Pol 1:500 rabbit anti-FMRFa (Chemicon/Incstar)	Embryos, alevins, fry	Developing terminal nerve, hyp (NAPv, NPPv)	Regulation of neural centers related to analgesia, feeding	(20)

FMRFa (NPFF + ?)	Lungfish (*Neoceratodus forsteri*)	ir	Pol 1:10000 anti-FMRFa, Phoenix	Just before hatching to juvenile stages	Paraventricular organ in hyp, terminal nerve at hatching	–	(21)

FMRFa (NPFF + ?)	Frog (*Rana esculenta*)	ir	Pol FMRFa antiserum (Peninsula labs)	Posthatching	tel and diencephalon (newly hatched)	Modulation of GnRH-neurons?	(22)

NPFF	African clawed frog (*Xenopus laevis*)	ir	Pol 1:1000 rabbit anti-NPFF serum (from Dr. H.Y.T. Yang, Elisabeth’s Hospital, Washington, DC, USA)	E30–45, and through metamorphosis	Olfactory bulbs and ventral tel, hyp, NTS, and spinal cord in embryo	Regulation of α-MSH release? Spinal embryogenesis?	(15)

FMRFa (NPFF + ?)	Toad (*Bufo bufo*)	ir	Pol 1:30000 rabbit anti-FMRFa (Phoenix)	Embryonic and larval stages	Suprachiasmatic area in embryo (stage III_6_). Olfactory bulb, tel, suprachiasmatic hyp in early larvae	Neuromodulator/neurohormone during development	(23)

FMRFa (NPFF + ?)	Skink (*Chalcides chalcides*)	ir	Pol 1:10000, 1:30000 anti-FMRF (Phoenix)	7 –70 dpf (birth)-neonatal	Fore- and hindbrain (terminal nerve, OB, hyp lateral preoptic area, suprachiasmatic area, and NAPv), MRF (35 dpf), plus NTS and vagus nerve close to birth	Regulation of blood pressure? Control of pituitary?	(24)

FMRFa (NPFF + ?)	Chicken (*Gallus domesticus*)	ir	Pol 1:4000 FMRFa antiserum (Peninsula labs)	E11–19	TN	–	(25)

FMRFa (NPFF + ?)	Japanese quail (*Coturnix japonica via*)	ir	1:5000 Anti-FMRFa (26), 1:3000 anti-FMRFa (Cambridge Research Biochemicals), 1:3000 anti-bovine F8F (2)	E2.5–12	Fibers in diencephalon (hyp), brain stem, olfactory nerve, and cell bodies in septum at early stages. OB at later stages	–	(16)

FMRFa (NPFF + ?)	African clawed frog	ir	Pol 1:1000 rabbit anti-FMRFa (Diasorin, Stillwater, MN)	Through metamorphosis	Olfactory nerve, tel, suprachiasmatic hyp (prometamorphic stage 56)	–	(27)

NPFF and receptors	Mouse	Quantitive autoradiography	Radioligand: [^125^ I](1DME)Y8Famide	Post-natal	Almost all brain areas at P14	Pro-opioid (P14) and anti-opioid effect (P21) of NPFF	(28)

NPFF	Rat	ISH, qPCR	–	E14-birth	Spinal cord, medulla (caudal NTS; E14), MRF (P0), pituitary	Sensory projection development in MRF, lactrotrope differentiation?	(18)

NPFF	Rat	ir	Pol rabbit anti rat F8Fa (FLFQPQRF)	E16, E18, E20, and post-natal	Fibers in median eminence (E20), cells in medulla (P1)	Role in homeostatic mechanisms, food intake in neonatals?	(17)

FMRFa (NPFF + ?)	Tree shrew (*Tupaia belangeri*)	ir on pituitary	Pol 1:1000 rabbit anti-FMRFa (Incstar, Stillwater, MN, USA)	E20–E41	Pars intermedia of pituitary from E27	Involved in early hormone secretion and releasing factor regulation?	(29)

FMRFa (NPFF + ?)	Tree shrew	ir	Pol 1:1000 rabbit anti-FMRFa (Incstar, Stillwater, MN, USA)	E19–E43	Developing TN from E23	–	(30)

*The brain areas are generally named according to the original article. α-MSH, α-melanocyte-stimulating hormone; dpf, days post-fertilization; E, embryonic day; hpf, hours post-fertilization; hyp, hypothalamus; ir, immunoreactivity; ISH, *in situ* hybridization; M, monoclonal; MRF, medullary reticular formation; NAPv, anterior periventricular nucleus; NPP, periventricular preoptic nucleus; NPPv, posterior periventricular nucleus; NTS, nucleus of the solitary tract in medulla; OB, olfactory bulb; P, post-natal day; Pol, polyclonal; qPCR, quantitative PCR; tel, telencephalon*.

### NPFF in adult vertebrates

In adult agnathans, NPFF RFa has been found expressed in the hypothalamus. Furthermore, it has been shown that NPFF stimulates the expression of the gonadotropin-β gene in the pituitary of hagfish, which suggests that NPFF can have a role in control of reproduction in lower vertebrates (31). In adult teleost fishes, NPFF seems to be exclusively expressed in gonadotropin releasing hormone 3 (GnRH3) neurons of the terminal nerve (TN) that stretches parallel to the olfactory nerve from the olfactory organ to the nucleus olfactoretinalis in the telencephalon (14). The TN, also known as the cranial nerve 0 (or N), was first described in sharks over 100 years ago, and later it has become clear that most vertebrates possess this nerve, from teleosts to primates, although the function is still not fully understood (32). One characteristic of these cells is that they express one variant of GnRH (32), which, as mentioned above, is GnRH3 in fish. The TN cell bodies are located parallel to the olfactory nerve, through the olfactory bulb to the telencephalon, but their axons project throughout the brain, affecting many different behaviors, especially reproduction-related behavior (33–35). Since it has been shown that NPFF can inhibit pacemaker activity of TN GnRH-cells, NPFF is believed to be involved in the regulation of reproductive behavior in fishes (36). In amphibians, NPFF-immunoreactive (NPFFir) cells have been identified in the preoptic area and hypothalamus (primarily the suprachiasmatic region), and extensive networks of NPFFir fibers are found throughout the brain, such as in the telencephalon, hypothalamus, medulla, and dorsal spinal cord (15, 37–39). In addition, many amphibians show NPFFir of the TN cells and fibers, similar to the findings in fishes. However, there seem to be species-specific variations regarding expression of NPFF in this site in amphibians (40). To our knowledge, the roles of NPFF in amphibians, reptiles, and birds are unknown. In rodents, NPFF cell bodies have not been identified in anterior brain regions, but are instead found in the hypothalamus, medulla, and spinal cord (41, 42). However, a dense network of NPFF fibers extends throughout most of the brain, and also to the pituitary, suggesting that NPFF can be involved in a range of different processes in mammals. In addition, there seem to be species-specific differences in the location of NPFF in mammals, since bovine cortex and hippocampus contains NPFFir (43). The known effects of NPFF are very diverse in mammals; most importantly NPFF has been found to act as a neuromodulator in the opioid system, but has also been found to increase arterial blood pressure, reduce water intake, inhibit the release of vasopressin from the neurohypophysis, and influence food intake (6, 44).

### NPFF in fish development

In zebrafish (*Danio rerio*), NPFF expression first appears already at 30 h post-fertilization (hpf; faringula period) in a small cell cluster just ventral to the olfactory placode (*in situ* hybridization) (14). The NPFF-positive cells co-express GnRH3, a marker of ganglion cells of the TN. These cells are also marked with FMRFa polyclonal antibodies, both in 30 hpf zebrafish embryos and juvenile sturgeon (sterlet, *Acipenser ruthenus*) (19), as well as in early embryos of trout (*Salmo trutta fario*) (20) and lungfish (*Neoceratodus forsteri*) (21). However, in the latter three studies, at least one additional cluster of cell bodies was marked with FMRFa immunoreactivity (FMRFir), situated in the diencephalon (more specifically, the periventricular hypothalamus in zebrafish and trout, circumventricular regions of hypothalamus in sterlet, and the paraventricular organ in lungfish). These cells were not labeled with *in situ* hybridization in zebrafish. Thus it seems that the FMRFa polyclonal antibodies bind one or several other RFa in addition to NPFF, making it challenging to interpret immunohistochemical data for FMRFa (14, 19). At 2 dpf, NPFF-expressing cells are breaking off from the small cell cluster near the olfactory placode and start caudal migration, forming a chain along the TN trajectory in zebrafish (*in situ* hybridization) (14). This pattern is also seen with FMRFa-immunohistochemistry in zebrafish, sturgeon, and lungfish (19, 21), and is proposed to visualize a migration route for cells of the TN from the olfactory placode to the nucleus olfactoretinalis in the telencephalon (19). The origin of the TN cells was believed to be the olfactory placode, but more recent studies done in zebrafish have shown that the cells of the TN originate from the neural crest and then invade the olfactory placode and later migrate from the olfactory placode area to anterior brain regions (45, 46). The hypothalamus and the spinal cord of the zebrafish did not show any NPFF-labeled cells with *in situ* hybridization, neither in the embryo nor the adult (14). This pattern seems to be in contrast to the pattern in other vertebrates, where NPFF-expressing cell bodies can be found in other brain areas during development (see NPFF in Amphibian, Reptile, and Avian Development and NPFF in Mammalian Development).

### NPFF in amphibian, reptile, and avian development

Similar to teleosts and lungfish, FMRFa-immunohistochemistry labels neurons of the TN of African clawed frog (*Xenopus laevis*), also during development. Using an anti-NPFF serum produced in rabbit, Lopez et al. (15) observed NPFFir cells in the embryonic olfactory placode, which is attached to the developing telencephalon at stage 40 in *Xenopus*. Later (stage 43), NPFFir cells could also be seen in the ventral part of olfactory bulb, and the developing telencephalon, rostral to the anterior commissure. Similar labeling of the developing TN is also found with less specific antibodies for FMRFa in *Xenopus* (27) and also in other amphibians, such as European green frog (*Rana esculenta*) (22) and toad (*Bufo bufo*) (23), in the reptile skink (*Chalcides chalcides*) (24), and in the birds Japanese quail (*Coturnix japonicavia*) (16) and chicken (25). Similar to zebrafish, the cells of the TN in these studies migrate from the olfactory placode, along the olfactory bulbs, to the telencephalon. Destruction of the olfactory placode in the toad embryo leads to elimination of FMRFa cells in the olfactory bulbs, ventral telencephalon, and anterior preoptic area, but not the cells in the hypothalamus (see next paragraph), showing that as in fish, the NPFF neurons of the TN migrate from the olfactory placode (23). Also in Japanese quail, the migration of FMRFa (F8Fa) neurons of the TN is similar to fish and amphibians (16), showing that this is an evolutionary conserved feature of the TN.

In contrast to the apparent situation in teleosts, the developing and the adult brains of amphibians and birds show presence of NPFF mRNA and protein also in brain areas other than the TN (15, 16). The main population of NPFFir cells in *Xenopus* embryos is found in the suprachiasmatic region in the hypothalamus, and these cells appear earlier than the neurons of the TN (15). This area projects to the intermediate lobe of the pituitary and is involved in the control of body color in *Xenopus* through the control of α-melanocyte-stimulating hormone (α-MSH) that stimulates the melanophores in the skin (47). The suprachiasmatic cells were immunoreactive very early in development (stage 30), before production of α-MSH starts, suggesting that NPFF may be involved in the control of melanotrope cell development (15). The hypothalamic neurons seem to innervate the tectum, torus semicirculris, and tegmentum in the mesencephalon, and the innervation increases during development in *Xenopus* (34). The suprachiasmatic region is also labeled with FMRFa antibodies early in embryonic *Xenopus*, toad, European green frog, and skink (22–24, 27, 39). Placodectomy studies in both birds and amphibians have shown that this population of cells has a different developmental origin than the FMRFir TN cells (16, 23, 39, 48).

Interestingly, neurons in the nucleus of the solitary tract in the medulla show NPFFir at an early stage in *Xenopus* (20). This area projects to the parabranchial region and the innervation is involved in the control of feeding in mammals (49). The cell bodies in the nucleus of the solitary tract seem to decrease in their immunoreactivity during development, but the projection to the parabranchial area persists and this area is heavily innervated with NPFFir fibers when the tadpoles start feeding (15). Also in developing mammals, NPFF cells are found in the nucleus of the solitary tract (see next section). At the climax of metamorphosis, NPFFir cells are detected in the reticular formation of the brain stem (15), also similar to findings in mammals. In the spinal cord, NPFFir elements were detected early in development, first in rostral spinal segments and later in the thoracic and upper lumbar segments. Interestingly, the NPFFir intensity was higher in the *Xenopus* spinal cord than in the adult, suggesting that NPFF has a developmental role in spinal cord embryogenesis. At the end of metamorphosis, the adult pattern of NPFFir structures in *Xenopus* is established (15). The brain stem and the spinal cord is not labeled with FMRFa antibodies in frog or toad (22, 23), but the skink shows FMRFir in these locations during development (24).

In the Japanese quail, the first NPFFir (F8F) fibers appear in the diencephalon (later hypothalamus) and the brain stem at embryonic stage (E) 6 (16). Later, also fibers in the olfactory nerve and the septum are labeled. In later developmental stages (E12), fibers and cell bodies are seen in the already mentioned areas in addition to the olfactory bulb. The location of NPFFir cells is similar to GnRHir cells.

### NPFF in mammalian development

Using an antibody against rat NPFF (F8F-amide), Kivipelto et al. showed the presence of fibers and terminal-like structures as early as E20 in the rat (*Rattus norvegicus*) (17). The labeling was seen in the internal layer of the median eminence in hypothalamus, an area important for control of pituitary hormone secretion. However, no labeled cell bodies were detected anywhere in the brain at this stage. At birth, NPFFir cells were found in the caudal part of the medial nucleus of the solitary tract in medulla, parallel to findings in *Xenopus*. As mentioned, this area is involved in control of feeding through its innervation to the lateral parabranchial nucleus (an area associated with feeding control) (50). In accordance with the findings in *Xenopus*, a relatively dense area of NPFFir terminals and fibers were found in the parabranchial nucleus in the rat (17). In addition, fibers and terminals were found in numerous other parts of the brain; for instance paraventricular hypothalamic area, supraoptic nucleus, optic decussation, and the periventricular hypothalamic area. Dense networks of fibers and terminals were seen in the internal layer of median eminence and infundibular stem. Further caudally, scattered fibers were found in the central gray and the inferior colliculus. At post-natal day (P) 3, also some cell bodies were found in the caudal spinal nucleus of the trigeminal nerve (which is the place all the pain and temperature fibers from the face terminate) and the dorsal horn of the spinal cord. By the age of 4 weeks, the distribution of immunoreactivity was similar to adults, where cell bodies could be seen in the periventricular area of the medial hypothalamus in addition to more intense labeling of the previously mentioned areas (17, 51).

In an *in situ* hybridization study on developing rat, Nieminen et al. (18) found expression of NPFF at E14, much earlier than the presence of NPFF protein seen with immunohistochemistry [at E20; (17)]. E14 embryos showed NPFF expression in the medulla and spinal cord (18). Later (E17), distinct neurons expressed NPFF in the spinal cord, and at birth NPFF expression was seen in neurons in the rostral nucleus of the solitary tract in the medulla, similar to what was found with immunohistochemistry. In addition, reticular nucleus (corresponding to lateral medullary reticular nucleus in adult rats) was found to express NPFF at birth, parallel to findings in developing *Xenopus* (15). This expression was transient in the rat, indicating that NPFF may be involved in development of the sensory trajectories passing through this nucleus (18). Expression of NPFF was also seen in the pituitary of the embryonic rat, but no NPFF-expressing cells were observed here. Finally, NPFF expression was found in the developing lung and spleen.

Using an NPFF radioligand, Desprat et al. showed the presence of receptors for NPFF in regions of the developing mouse brain and spinal cord involved in the analgesic effects of opiates (28). During post-natal development, they found that NPFF affected the morphine-induced analgesia in different ways in the neonatal, but in adults NPFF had only anti-opioid effect. From birth, they could detect binding of NPFF in the olfactory bulb, and from P7 in the ventral pallidum and nucleus ventral endopiriform in the telencephalon. Also in the diencephalon in the nucleus reuniens NPFF binding appeared at P7, and in the mesencephalon a few binding sites was visible at birth. This study shows that the interplay between NPFF receptors and opioid receptors is established at early stages in mice. However, embryonic stages were not studied, so the pattern of NPFF binding in the early developing mouse is not known.

Interestingly, the TN of the mammal tree shrew (*Tupaia belangeri*) shows FMRFir (30). This mammal is closely related to primates. The FMRFir pattern was similar to the pattern in fish, amphibians, reptiles, and birds, with FMRFa cells appearing early in embryogenesis (E20) near the olfactory epithelium, and later along the migrating route for the TN. Thus, it seems that NPFF expression in the TN is a feature conserved from fishes to mammals.

In summary, NPFF is detected early in embryonic development in all vertebrates studied, see overview in Table 1. In fishes, it seems to be exclusively expressed in the TN, also during development. In amphibians, reptiles, birds, and mammals NPFF can in addition be found in the suprachiasmatic region in hypothalamus at very early developmental stages. In *Xenopus* and mammals, medullary reticular formation also shows NPFF labeling in embryos. No function has been demonstrated for NPFF during development, but its expression pattern in the brain may suggest it could be involved in development of neurons of the TN, and nerve circuits involved in control of feeding.

## PrRP Group

The PrRP group includes the peptides PrRP31 and PrRP20. A new member of this family, C-RFa is found in Japanese crucian carp (*Carassius cuvieri*), zebrafish, *Xenopus*, and chicken (*Gallus gallus*), but this variant is not found in mammals (52). PrRP peptides bind the receptor GPR10 (prolactin-releasing hormone receptor; PRLHR; also named GR3), but they also bind NPFFR2. Three different receptors for PrRP peptides exist in some vertebrates, while only one is found in mammals [see receptor synteny in Wang et al. (52)].

### PrRP in adult vertebrates

It is believed that PrRP is involved in the control of pituitary function in fishes. Firstly, in many species of adult fishes, PrRP fibers project to and terminate on prolactin-producing cells of the pituitary. Secondly, C-RFa injections in rainbow trout and tilapia cause release of prolactin and somatostatin (53–56). Furthermore, PrRP cell bodies are found in the nucleus lateralis tuberis pars posterioris in guppy (*Poecilia reticulata*), rainbow trout (*Oncorhynchus mykiss*), and goldfish (*Carassius auratus*) (52, 56, 57), an area suggested to be important for control of pituitary function in teleosts (58). There seems to be some variation in PrRP expression between species. Some fish species have PrRP cell bodies also in other brain areas, while others do not seem to have PrRP fibers projecting to the pituitary (57).

In mammals, PrRP was thought to act on the pituitary, because of the high expression of its receptor GPR10 in the anterior pituitary (59). Preliminary studies in rats showed that the peptide could promote prolactin release from pituitary cells and from these studies the peptide got its name (60). However, later studies have shown that this pathway may not be physiologically relevant in mammals. Instead, it has been shown that PrRP is involved in control of food intake and energy balance in rats and mice, and that it can affect the stress response by elevating circulating levels adrenocorticotropic hormone (ACTH) (59). Further, it has been shown that PrRP has an effect on the cardiovascular system and on circadian rhythms in mammals. Interestingly, it has been suggested that endogenously produced PrRP peptide has an autocrine role in cell-cycle progression and growth (61), processes that are closely linked to development. PrRP is expressed in the nucleus of the solitary tract, ventrolateral medulla, and in the caudal portion of the dorsomedial hypothalamic nucleus in adult mammals (59). PrRP fibers are found in many areas of the forebrain, as in preoptic area, periventricular nucleus of the thalamus, and in periventricular nucleus and paraventricular nucleus of hypothalamus (62). In contrast to the situation in fish and amphibians, no PrRPir can be detected in the median eminence or in the hypophysiotropic cells of the hypothalamus (63, 64). In peripheral tissues, PrRP mRNA is found in the adrenal gland, pancreas, placenta, and testis (59).

### PrRP in vertebrate development

Very few studies have looked at the possible role of PrRP in development. However, the few that exist show that this peptide is expressed at an early stage in *Xenopus* (65), chicken (52), and rat (18, 62).

In the teleost guppy, PrRPir cells were detected in the nucleus lateralis tuberis pars posterioris in the hypothalamus already at the day of birth (57). However, innervation of PrRP fibers to the prolactin cells of the pituitary was not seen at birth day, but appeared later. Earlier developmental stages were not investigated, so the role of this peptide in teleost development is not clear.

In *Xenopus*, the expression of PrRP mRNA is detected at an early stage (stage 54; measured with qPCR) (65). PrRP mRNA levels were highest in early premetamorphic stages (stage 57), and decreased during prometamorphosis. This coincides with prolactin starting to appear in the pituitary. In the chicken pituitaries, the PrRP receptor PrRPR1 was expressed at E8 (measured with qPCR) (52), and the expression increased at later developmental stages (E12, E16, and E20). However, earlier stages were not studied.

In rat, PrRP mRNA and PrRPir cells are found in the nucleus of the solitary tract at E18, and in the ventral and lateral reticular nucleus of the caudal medulla oblongata at E20 (62). The hypothalamus first showed PrRP expression and PrRPir at P13. Similar to sexually mature rats, P6 animals had PrRPir fibers in paraventricular hypothalamic nucleus, periventricular hypothalamic nucleus, medial preoptic area, basolateral amygdaloid nucleus, dorsomedial hypothalamus, ventromedial hypothalamus, periventricular nucleus of the thalamus, and bed nucleus of the stria terminalis. However, also areas not showing PrRPir in the adult had PrRP in the developing rat brain at P6 and P9, like optic chiasm, dorsal endopiriform nucleus, cingulum, intermediate reticular nucleus, and caudal ventrolateral reticular nucleus. This transient expression could indicate a role in development of these brain areas.

The presence of mRNA of PrRP and its receptor GPR10 has also been investigated with *in situ* hybridization in rat embryos in a different study. Nieminen et al. (18) found a similar pattern as shown with immunohistochemistry. However, they found expression of PrRP in the reticular formation at a much earlier stage [E17 compared to P6 in (62)]. The receptor GPR10 was expressed at very early stages; at E15 in the pallium, at E16 in the hippocampus, and at E19 in the reticular formation. Interestingly, this is before any PrRP mRNA can be detected in these locations (18, 62), which may suggest that the receptor has an alternative ligand. In the pituitary, the expression of both PrRP and GPR10 starts at E18, at the same time as the lactotrops first appear (18). This is the opposite of the situation in *Xenopus*, where PrRP expression decreases when prolactin starts to appear in the pituitary (65). In the periphery, PrRP expression was seen in the developing liver, and to some extent in the spleen and kidney (18).

Studies of PrRP in vertebrate development are summarized in Table 2.

**Table 2:** **Overview of studies of PrRP in vertebrate development**.

RFa and/or receptors	Species	Method	Antibody	Embryonic stages	Location of peptide/mRNA in early developing CNS	Putative functions in early development	Reference
PrRP	Guppy (*Poecilia reticulata*)	ir	Pol rabbit anti-salmon PrRP (56)	0-P14	Hyp, pituitary pars distalis at birth	Developmental role?	(57)

PrRP	*Xenopus laevis*	qPCR	–	Premetamorphosis- climax (54–65)	Transiently increased expression in brain at prometamorphosis	–	(65)

PrRP	Chicken pituitary	RT-PCR	–	E8–20	Expressed in pituitary at all stages studied	–	(52)

PrRP	Rat	ISH, RT-PCR, ir	M 40 μl/ml P2L-1C (mature PrRP)/P2L-1T (prepro-PrRP) mouse anti human PrRP (66)	E15, E18, E20, and post-natal	NTS (E18), MRF (E20), hyp (P13)	Role in embryonic brain development?	(62)

PrRP + GPR10	Rat	ISH, qPCR	–	E14-birth	PrRP: MRF, pituitary (E19), GPR10: pallidum, hippocampus, and MRF (E15–17)	Lactrotrope differentiation?	(18)

*The brain areas are generally named according to the original article. E, embryonic day; hpf, hours post-fertilization; hyp, hypothalamus; ir, immunoreactivity; ISH, *in situ* hybridization; M, monoclonal; MRF, medullary reticular formation; NTS, nucleus of the solitary tract in medulla; P, post-natal day; Pol, polyclonal; qPCR, quantitative PCR; RT-PCR, reverse transcription PCR*.

## GnIH Group

GnIH was first described by Tsutsui et al. (67). This RFa was found to inhibit gonadotropin release in the quail through binding to the G-protein-coupled receptor GPR147. It has since been found that in birds, GnIH can act directly on gonadotrope cells to inhibit both synthesis and release of gonadotropins, e.g., Ref. (68–70). Moreover, it may also act on GnRH-neurons to inhibit GnRH release and thereby indirectly inhibit gonadotrope cells [e.g., Ref. (71)]. Since its first discovery in birds, GnIH orthologs have been found in most vertebrate classes [see review by Tsutsui and Ubuka (72) and references therein]. Alternative names are sometimes used in different vertebrate classes – the mammalian ortholog being named RFa-related peptide (RFRP) with the RFRP gene encoding two bioactive peptides, RFRP-1 (or NPSV) and RFRP-3 (or NPVF) (73). The mammalian receptor is sometimes referred to as NPFFR1 or NPFF1. In amphibians, GnIH is sometimes referred to as GRP, GRP-RPs, or R-RFa [e.g., Ref. (74)], while in teleosts the term LPXRFa may be used [e.g., Ref. (75)]. In the following, we will use the common name GnIH regardless of vertebrate class.

### GnIH in adult vertebrates

In adult vertebrates, GnIH positive cells are found in different regions of the brain, notably in hypothalamic regions like the avian paraventricular nucleus, from where they send their projections to GnRH1 neurons in the preoptic region or to gonadotrope cells in the pituitary. GnIH terminals and GnIH receptors have also been identified on GnRH2 neurons in birds and mammals, e.g., Ref. (71, 76). A recent paper shows that GnIH inhibits socio-sexual behavior of male quail through a direct activation of aromatase and thereby increased neuroestrogen synthesis in the preoptic area (77). Both GnIH and its receptor are also expressed in the pituitary in different vertebrate classes, indicating auto- or paracrine regulation [e.g., Ref. (78)]. In addition, various studies have revealed GnIH positive cells in the gonads (both testis and ovary), while PCR experiments have revealed gene expression in peripheral tissues like muscle, spleen, eye, and kidney [e.g., Ref. (79)]. The expression of both ligand and receptor in the avian gonads (80) again points to an auto- or paracrine role during gametogenesis.

The spatial expression pattern should indicate potential functions, although much remains to be discovered when it comes to GnIH functions in general and during development in particular. Similar to in birds, GnIH in mammals have been shown to inhibit gonadotropin synthesis and release, either directly in the pituitary or via inhibition of hypothalamic GnRH-neurons. The situation seems different in frogs and teleost fish where GnIH can either inhibit or stimulate gonadotropin (and also growth hormone and prolactin) release, depending on reproductive stage, species, and sex, e.g., Ref. (74, 79, 81–84). Also in an agnathan species (sea lamprey; *Petromyzon marinus*), GnIH stimulates expression of GnRH and gonadotropin β-subunit (85), indicating that this neuropeptide may have experienced a shift in function during vertebrate evolution.

As most interest has focused on its role as an inhibitor of GnRH and gonadotropin release during reproduction, very little is known about GnIH during vertebrate development. Apart from some studies looking at pre-pubertal stages, the information we have is mostly limited to studies on the spatio-temporal expression pattern in mammalian and avian (post-natal) development, and some very few in teleost early development.

### GnIH in vertebrate development

A recent article from Biswas and colleagues (78) provided some interesting information regarding the spatial expression pattern of GnIH peptides in Indian major carp (*Labeo rohita*), although detailed origin of their antibodies are missing from the paper. For instance, they found GnIH expression in the olfactory system (epithelium and bulb) in newly hatched larvae, indicating a non-reproductive function. Expression in hypothalamic nuclei such as the periventricular preoptic nucleus and the posterior periventricular nucleus, usually related to gonadotropin regulation in the adult, were also found in newly hatched larvae of the Indian major carp. Moreover, GnIH was found expressed in the anterior part of the carp pituitary [rostral pars distalis (RPD) and proximal pars distalis (PPD)] already from hatching, although no staining could be found at the adult stage, again pointing to an autocrine or paracrine function. Another recent study provides information of the temporal gene expression pattern of GnIH and its receptors during zebrafish early development (79). Whereas each of the three GnIH receptor paralogs could be found expressed (RT-PCR) already at the blastula stage and all the way through to the adult stage, although with differential expression patterns, the GnIH ligand was found only from a later embryonic stage; at 24 hpf (early pharyngula period). Nevertheless, these results indicate a functional GnIH ligand/receptor system active already from early embryonic stages in zebrafish. This is supported by recent results from our own lab, where we find expression of medaka (*Oryzias latipes*) *gnih* and *gnihr1* already from 1 hpf and throughout the larval period, indicating maternal transfer in medaka (own unpublished data). The other two receptor paralogs in medaka, *gnihr2* and *gnihr3* were also expressed from early stages and throughout the larval period, although not until after the mid-blastula transition. The expression profile of medaka *gnih* resembled that seen in GnIH neurons in post-natal mice (86) with an initial increase followed by a steady decrease in expression levels. There is no existing data on the spatial expression of GnIH ligand or receptor during early development in fish.

In birds, where GnIH was first characterized, the existing literature focuses on the function of the GnIH system during sexual development, especially during the pre-pubertal period. For instance, circulating gonadotropin levels have been found to be negatively correlated with hypothalamic GnIH content (87). In immature male quail, chronic injections of GnIH suppressed normal testicular development, including reduced plasma testosterone levels and suppression of germ cell proliferation and seminiferous tubule development (70).

As mammalian model systems are less suited for studies of early embryogenesis, the few papers dealing with GnIH in mammalian development starts from late gestational stages. Yano et al. (88) found both GnIHs (RFRP-1 and -3) expressed in rat fetal hypothalamus from E15–E16 (mRNA) and embryonic day 16–17 (protein), showing first evidence of the existence of a functional system at the fetal stage also in mammals. Using a combination of GnIH *in situ* hybridization and BrdU immunohistochemistry, Legagneux et al. (89) identified GnIH producing neurons in male and female rat exclusively in the tuberal hypothalamus. These neurons to a large extent developed early, around E13–E14. In male and female rats, both Quenell et al. (90) and Iwasa et al. (73) found progressive gene expression of both GnIH and its receptor from early post-natal stages (from 4 days) all the way through puberty to the adult stage. Iwasa et al. also measured GnIH peptide levels and found a profile similar to that of GnIH gene expression, suggesting that the GnIH system indeed is active and plays a role during sexual development in rats (73). In accordance with these results, Poling et al. (86) also detected early gene expression of GnIH. mRNA levels increased in both sexes during post-natal and pre-pubertal development, before a decline was seen between post-natal day 20 and adulthood. During development in male rats, sustained knockdown of GnIH led to increased plasma levels of LH and increased testicular growth (91). These data indicate that GnIH act as inhibitor of gonadal maturation and puberty, similar to the situation in birds. However, Iwasa et al. (73) found increased GnRH gene expression levels concomitant with increased GnIH ligand and receptor expression during development in rat. This suggests that GnIH alone is not sufficient as inhibitor, but that regulation of sexual development is more complex, probably including additional factors such as other RFa, in addition to energy-related factors like ghrelin or leptin, or other.

In summary, the expression of a seemingly functional GnIH system in fish, birds, and mammals already from early development suggests important developmental function(s) of this RFa in vertebrates. If these include more than the above mentioned regulatory (inhibitory/modulatory) effects on sexual development, remains to be seen. See Table 3 for an overview over developmental studies of GnIH.

**Table 3:** **Overview of studies of GnIH in vertebrate development**.

RFa (and/or receptors)	Species	Method	Antibody (or radioligand)	Embryonic stages	Location of peptide/mRNA in early developing CNS	Putative functions in early development	Reference
GnIH	Indian major carp (*Labeo rohita*)	ir	?	Hatchling-fry-juvenile	Cells in olfactory system, NPP, NPPv, and fibers in optic tectum, PPD in pituitary, and MRF (P0)	–	(78)

GnIH + receptors	Zebrafish (*Danio rerio*)	RT-PCR	-	Blastula-juvenile	GnIH first detected at 5-prime stage, receptors at all stages	Role in early development?	(79)

GnIH	Rat	ISH, RT-PCR, ir	M 10 μg/ml 1F3 anti-RFRP-1, P 16 μg/ml antisera anti – FRP-1 (92, 93)	E15, E18, E20, and post-natal	Caudal portion of hyp (E16), many areas at E18 and E20	Modulation of pain, response to stress during development?	(88)

GnIH and GPR147	Rat	qPCR,ELISA	Pol rabbit anti-avian GnIH (67)	Pre-pubertal (P4–20) and peripubertal	GnIH and receptor mRNA and peptide present in hyp from P4	–	(73)

GnIH	Rat	ISH + BrdU	–		Cell bodies generated at E13/E14 in tuberal hyp	–	(89)

GnIH	Mouse	ISH	–	P1, P10, P20	mRNA and protein in dorsal-medial nucleus of hyp from P1	–	(86)

*The brain areas are generally named according to the original article. E, embryonic day; hyp, hypothalamus; ir, immunoreactivity; ISH, *in situ* hybridization; M, monoclonal; NPP, periventricular preoptic nucleus; NPPv, posterior periventricular nucleus; P, post-natal day; Pol, polyclonal; PPD, proximal pars distalis in adenohypophysis; qPCR, quantitative PCR; RT-PCR, reverse transcription PCR*.

## Kisspeptin Group

Kisspeptins are RFa encoded by the *Kiss* gene. The resulting protein is further processed into bioactive peptides of variable lengths (94), while their receptors belong to the rhodopsin family of G-protein-coupled receptors (95–98).

### Kisspeptin in adult vertebrates

The product of the *Kiss* gene was first discovered as a metastasis suppressor and therefore termed metastin (99). However, kisspeptins and their putative receptors (Kissr or Gpr54) have during the last decade emerged as major gatekeepers of reproduction because of their central role in regulating the brain–pituitary–gonadal (BPG) axis [reviewed by Ref. (100)]. The importance of the Kiss system as a regulator of the BPG-axis came after observations that mutations in the *Gpr54-1* lead to idiopathic hypogonadotropic hypogonadism (101, 102). Besides its role as a tumor suppressor and regulator of the BPG-axis, several studies report additional roles of the Kiss system, including vasoconstriction (103, 104), neuronal migration, and increased synaptic transmission (105, 106). For example, Fiorini et al. (107) showed that stimulation with KISS increased neurite growth in GnRH-positive neurons *in vitro*. Although detailed mechanisms of action are still lacking, these seemingly pleiotropic roles may reflect the diversity of intracellular signaling pathways that can be triggered by Kiss receptor activation (100, 108). The current understanding in mammalian systems suggests that major endogenous and environmental signals act through Kiss neurons, which then directly or indirectly provide an integrated signal to the hypophysiotropic GnRH neurons.

### Kisspeptins during development

Despite the accumulating data of the role of kisspeptins in adult vertebrates, less is known about kisspeptins during post-natal/pre-pubertal development, and very little is known regarding the potential expression and function of the kiss system during embryogenesis/early development. This could at least partly be due to the lack of a suitable model system. Because *Kiss* or *Gpr54* KO mice are infertile, homozygous offspring need to be established from heterozygous parents. This means that the possibility of maternal transfer of transcripts, including those of *Kiss* and *Gpr54*, cannot be excluded in this system. Furthermore, and common to all RFa families discussed here, studying embryonic development in mammals *in vivo* is difficult due to their viviparity.

**Figure 1:**
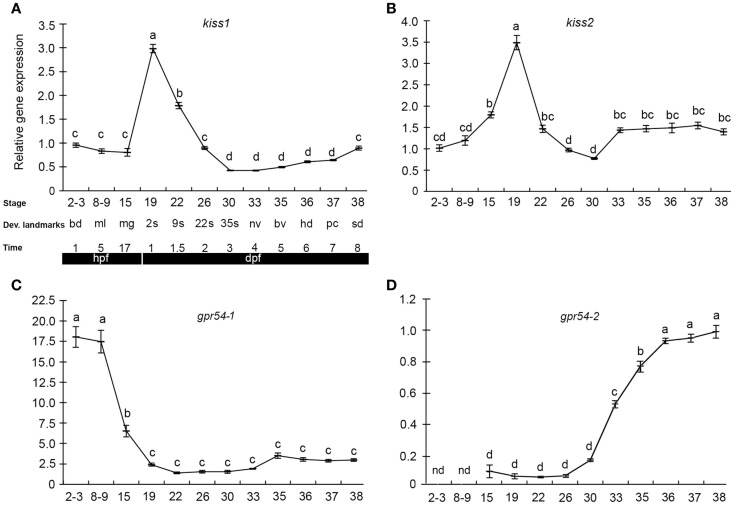
**Kiss and kiss receptors are expressed at very early stages in medaka embryos**. Relative gene expression of *kiss1* **(A)**, *kiss2* **(B)**, *gpr54-1* **(C)**, and *gpr54-2* **(D)** was analyzed at different developmental stages (mean ± SEM; *n* = 7). Key developmental stages are given above the age of the hours (h) or days (d) post-fertilization (hpf). The gene expression levels are given relative to a reference gene (β-actin). Different letters indicate significant differences (*P* < 0.05). Figure from Ref. (109).

The few existing data on kisspeptins during early development come from studies in medaka and zebrafish. We recently performed a study of kisspeptin ligand and receptor expression pattern and function during early development in medaka, exploiting the advantages of the teleost model system (109). qPCR gene expression profiles (Figure 1) revealed maternally provided Kiss systems involving the two *kiss* ligands (*kiss1* and *kiss2*) and one of the receptors (*gpr54-1*), indicating the possibility of functional Kiss receptor-ligand systems at very early stages. *gpr54-2*, on the other hand, was not detected until after the zygotic phase, at stage 15, with a significant increase in expression levels between stage 30 and stage 36. In zebrafish, *kiss1* and *kiss2* gene expression was reported in 24 hpf (30 somite stage) embryos (110), but earlier stages were not investigated. In another teleost, the cobia (*Rachycentron canadum*) *gpr54-1* expression was detected at 1 day post hatching (111). The early expression of *kiss* and *gpr54* also coincides with the early expression of gonadotropins in fish (112), indicating a potentially functional BPG-axis already during early embryogenesis.

**Figure 2:**
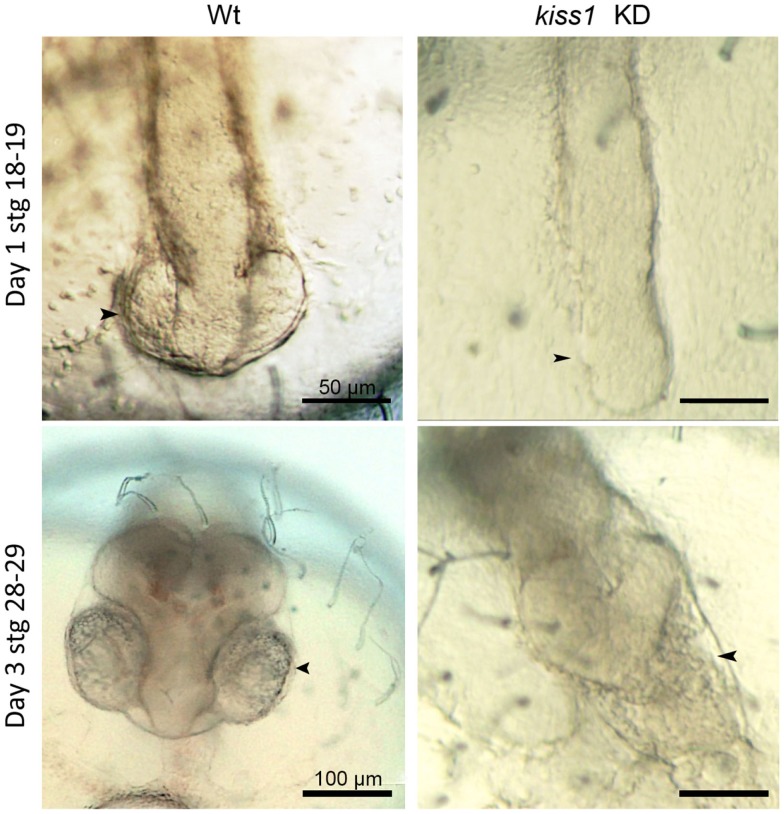
**Low dose antisense knockdown (KD) lead to impaired brain and eye development**. *kiss1* knockdown embryos showed impaired eye development at 1 dpf (top right). At 3 dpf, there was no development of the forebrain after *kiss1* knockdown (bottom right). Scale bars correspond to 50 μm (top images) or 100 μm (bottom images). Arrowheads point to the area of the developing eyes. Figure from Ref. (109).

In Hodne et al., we performed a series of knockdown experiments that indicated several independent kiss systems during medaka embryonic development (109). Both maternally and zygotically expressed *kiss1* and *gpr54-1* seemed critical for proper development (Figure 2). However, the apparent functions of the maternally and zygotically expressed transcripts were quite distinct, as explained below.

Knockdown of maternal *kiss1* and *gpr54-1* led to developmental arrest and subsequent death around the blastula stage (stage 10–11), suggesting that this early expressed system could be involved either in regulation of early asynchronous cell division or early cell migration. The downstream factors controlled by *kiss1*/*gpr54-1* signaling are not known. However, cell migration during blastulation and gastrulation is dependent on *sdf1*/*cxcr4* chemotaxis. This signaling pathway is also known to be important during bone-directed migration of GPR54-positive breast cancer cells (113) and kisspeptin can indirectly regulate *sdf1*/*cxcr4* through desensitization of *cxcr4* by preventing rise in intracellular Ca^2+^ levels after *sdf1* stimulation (114, 115). In zebrafish, knockdown of *sdf1*/*cxcr4* inhibits migration of endodermal cells during gastrulation (115). Moreover, *sdf1* signaling is crucial for survival in mice, and individuals lacking either receptor or ligand have defective hematopoiesis, developmental lymphoid tissue, vascularization of gastrointestinal tract, migration of neuronal cells, and patterning in the central nervous system, and they die prenatally (116, 117).

Zygotic knockdown of *kiss1* and *gpr54-1*, on the other hand, allowed the embryos to survive gastrulation and a seemingly normal development continued until completion of neurulation (stage 18). At this point, early eye development is normally observed. However, after zygotic knockdown with either morpholino or low dose of peptide nucleic acid (PNA), eye development was interrupted, and further brain development was severely disrupted (Figure 2). Surprisingly, knockdown of *kiss2*, which was expressed at similar levels as *kiss1*, did not produce any increased mortality or malformed embryos. Unless *kiss1* takes over the role of *kiss2* following *kiss2* knockdown, *kiss2* does not seem to be critical for proper development. If *kiss2* is translated and active before zygotic activation, our results indicate that it does not work through *gpr54-1* (which is not expressed at this point), but possibly through *gpr54-2* (although knockdown of this receptor leads to developmental arrest and 100% mortality), or through other, currently unknown, RFa receptors. The different effects observed following *kiss1* and *kiss2* knockdown suggest the possibility of a very early separation of two functional systems during embryonic development. One system, comprised of Kiss1 and Gpr54-1, has a functional role important for survival during the maternal stage of development. This system continues to function throughout embryonic development, although it seems more important for regulating brain development at later embryonic stages. A second system seems to be comprised of Kiss2 binding to either Gpr54-2 or other unknown RFa receptors. The possible function of this second system remains to be clarified.

Contrary to the observed phenotypes following zygotic knockdown of *kiss1* and *gpr54-1* outlined above, a moderate knockdown of medaka *gpr54-2* arrested further development at stage 16 (late gastrula). This phenotype resembled that of maternal *kiss1* and *gpr54-1* knockdowns. However, as *gpr54-2* is first detected after transition to zygotic gene expression, new questions arise as to why a similar phenotype was not also observed after zygotic *kiss1* and *gpr54-1* knockdown. One explanation could be that the two receptors are functionally separated. If they are involved in similar functions, our results indicate that the actions of Gpr54-1 may be partly compensated for by Gpr54-2, whereas Gpr54-2 cannot be functionally replaced by Gpr54-1.

A recent work by Zhao et al. has investigated the role of kiss on GnRH neuron development in zebrafish (118). In line with Kitahashi et al. (110), *kiss1* and *kiss2* mRNA could be detected by qPCR from 24 hpf. Furthermore, it was shown that both kiss ligands stimulated GnRH3 neuron proliferation peripherally, while only *kiss1* stimulated proliferation and synaptic contact points of GnRH3 neurons in the TN and hypothalamic regions.

The existence of a functional kisspeptin system in birds is not clarified [see in Ref. (119)], and consequently, there are no data on this during bird development.

In mammals, the kisspeptin system has been intensively investigated during the last decade. Whereas, most literature covers the key role of Kiss in regulating GnRH neuron around and after puberty, pre- and early neonatal stages have been looked into more closely during recent years [see reviews in Ref. (120, 121) and references therein]. For instance, *Kiss1* gene expression has been detected in hypothalamic areas during the late fetal period in mice [stage E13.5; (122)] and both gene expression and peptide have been detected in rats [stage E14.5, (123)]. There are two hypothalamic areas expressing *Kiss*; the arcuate nucleus (ARC), and the preoptic AVPV (anteroventral periventricular nucleus) and PeN (rostral periventricular nucleus). Whereas, Kiss neurons appear in the ARC prenatally, Kiss expression in the AVPV/PeN is not seen until neonatal stages. There seems to be clear sex differences in the expression of Kiss in rodents, with females expressing higher levels than males in both the ARC and the AVPV/PeN. *Gpr54-1* gene expression has also been detected in stage E13.5 prenatal mice (122), indicating the possibility of a functional ligand–receptor system from this stage on. Also in second trimester human fetuses, KISS and GPR54 immunoreactivity was detected in the hypothalamus. Maternally provided Kiss ligands or receptors have, however, not been reported.

It seems that the early kisspeptin systems are functional in rodents in that Kiss neurons already are in close contact with GnRH-neurons prenatally, and that GnRH-neurons are able to respond to kisspeptins by enhanced GnRH secretion during prenatal life (120, 122, 124, 125). Based on these and several other studies, a more generalized picture is starting to appear with a seemingly functional kisspeptin system in place during the last part of gestation. The Kiss neurons increase in number and activity and reach a peak prenatally, before a decreased activity around birth, and then a new increase again during early neonatal life before the activity decreases to low levels until the pre-pubertal stage. The prenatal and early neonatal peaks in Kiss neuron activity seemingly coincide with similar peaks in GnRH and pituitary gonadotropin secretion [see in Ref. (120, 121, 126–132)]. However, the function of this early expression of Kiss remains elusive.

In line with the more severe phenotypes observed in medaka following receptor knockdown (109), Lapatto et al. (133) also described a more severe phenotype following *Gpr54-1* knockout- compared with *Kiss1* knockout mice. As mice possess only the one Gpr54 paralog, one of several suggestions was a possible weak activation of Gpr54-1 by other ligands. The results of Hodne et al. (109) and those of Lapatto et al. (133) suggest that Kiss and possibly other RFa may promiscuously bind to different RFa receptors [see also in Ref. (134–136)]. Interestingly, Mayer and Boehm (137) found that female mice with genetically ablated kisspeptin neurons underwent puberty and became fertile. In contrast, acute ablation in adult mice inhibited fertility. These results clearly indicate compensatory mechanisms for early loss of kisspeptins. Whether maternally transferred kiss is crucial for mouse development has not been investigated.

Although more data are available regarding the role of kisspeptins during vertebrate development compared to the role of other RFa, there are still much work to be done. One important aspect probably will be to elucidate their role in neuronal migration/development, where they seemingly play a major role, at least in fish. See Table 4 for an overview over developmental studies of kisspeptins.

**Table 4:** **Overview of studies of kiss in vertebrate development**.

RFa (and/or receptors)	Species	Method	Antibody	Embryonic stages	Location of peptide/mRNA in early developing CNS	Putative functions in early development	Reference
Kiss	Zebrafish (*Danio rerio*)	qPCR, kiss treatment, electrophysiology	–	1–7 dpf	Kiss1 and 2 mRNA detectable in brain from 1 dpf, increasing during development	Kiss1 stimulates GnRH neuron development, Kiss2 involved in development of trigeminal neurons	(118)

Kiss	Zebrafish	qPCR	–	1, 3, 7, 30, 45 dpf, adult	*kiss1* and *kiss2* detected from 1 dpf	–	(110)

GPR54	Cobia (*Rachycentron canadum*)	qPCR	–	Post hatching-adult	*gpr54* present at all stages	–	(111)

Kiss and receptors	Medaka (*Oryzias latipes*)	qPCR+ knockdown	–	From fertilization to newly hatched	–	Essential for brain and eye development	(109)

Kiss	Rat	qPCR, ISH ir, BrdU birth dating	Pol sheep anti-kiss (N-ter) AC067	Embryonic rats from E11.5 to E21.5	Kiss1 neurons in arcuate nucleus born from E12.5	Involved in embryonic activation of the hypothalamic–hypophyseal–gonadal axis	(123)

Kiss	Rat	ISH	–	Post-natal (neonate to adult)	Anteroventral periventricular nucleus (P7 in males, P21 in females), arcuate nucleus (P3)	–	(131)

Kiss	Rat	ISH	–	Post-natal (P0–P19)	Anterior hyp (P11), arcuate nucleus (P0)	Role in sexual differentiation of neonatal brain	(130)

Kiss	Rat	Kiss stimulation (*in vivo* and *ex vivo*)	–	Post-natal	–	Stimulating GnRH release in neonatals (5P)	(126)

Kiss + GPR54	Rat	qPCR on hyp	–	Post-natal (P1–75) + adults	*Kiss* and *Gpr54* present at all stages	–	(138)

Kiss + GPR54	Mouse	Transgenic mice	–	E12.5, E13.5, E14.5, and E16.5	*Kiss*: arcuate nucleus in hyp (E13.5), *Gpr54*: restricted to GnRH-neurons in anterior forebrain (E13.5-post-natal)	Regulating fetal GnRH activity?	(124)

Kiss + GPR54	Mouse	RT-PCR, kiss treatment++		E12.5, E13.5, E14.5, and E15.5	*Kiss1*: mediobasal hyp (E13.5), *Gpr54*: preoptic area (E13.5)	Stimulates GnRH neurite growth	(107)

Kiss + GPR54	Mouse	ISH, qPCR, ir	Pol 1:10000 rabbit anti-rodent-kiss 1 (139)	E13, E15, E17 to P35	*Kiss1*: near median eminence (E13); preoptic periventricular nucleus (P12), *Gpr54*: from nasal compartment to forebrain in migrating GnRH-neurons (E13)	Involved in sexual differentiation of the brain during embryonic development?	(132)

GPR54	Mouse	ISH, single cell qPCR, Ca^2+^ imaging	–	E12.5, E13.5, E14.5, E17.5, and adult	In GnRH-cells in nasal region and nasal forebrain junction (E13.5)	–	(122)

Kiss	Mouse	ir	Pol 1:5000 rabbit anti-kisspeptin-10 (no. 566) (139)	Post-natal-adults (P10–P61)	Anteroventral periventricular nucleus, preoptic periventricular nucleus in hyp (P25) and arcuate nucleus in hyp at all stages	Kiss neurons in anteroventral periventricular nucleus and preoptic periventricular nucleus in hyp involved in the sexually differentiated functioning of GnRH-neurons	(127)

Kiss	Mouse	ISH	–	Post-natal (P1–P16)	Anteroventral periventricular nucleus and preoptic periventricular nucleus in hyp from P10	Involved in the sexually differentiated functioning of GnRH-neurons	(129)

Kiss	Mouse	ir	Pol 1:10000 rabbit anti-kisspeptin-10 (no. 566) (139)	Post-natal (P15–P30) + adults	Preoptic periventricular nucleus in hyp from P15	–	(128)

*The brain areas are in general named according to the original article. dpf, days post-fertilization; E, embryonic day; hpf, hours post-fertilization; hyp, hypothalamus; ir, immunoreactivity; ISH, *in situ* hybridization; P, post-natal day; Pol, polyclonal; qPCR, quantitative PCR; RT-PCR, reverse transcription PCR; tel, telencephalon*.

## 26RFa/QRFP Group

The 26RF/QRFP group is the newest member of the RFa family, first described in 2003 in the brain of European green frog (140). The gene for 26RFa/QRFP is found in genomes of many species, from teleost fish to human, with preserved synteny in human, mouse, chicken, and *Xenopus* (4). Mature peptides generated from this gene are 26RFa, 43RFa in rat, mouse, human, and frog, 9RFa in human and frog, and 26RFa and 7RFa in fish (4, 141). 26RFa/QRFP binds the receptor GPR103/26RFaR (142, 143). In addition, the peptide has affinity for NPFFR2 (144).

### 26RFa/QRFP in adult vertebrates

In adult goldfish, *26RFa/qrfp* mRNA is found in the hypothalamus, optic tectum-thalamus, and testis (141). Because the expression in the hypothalamus was significantly reduced after 4 days of starvation, and intraperitoneal injections of the 26RFa/QRFP peptide increased LH levels, 26RFa/QRFP has been suggested to play a role in the regulation of energy homeostasis and regulation of the BPG-axis in fish (141). Also in birds, 26RFa/QRFP is expressed in the diencephalon; in the anterior hypothalamic nucleus in chicken, and in the anterior-medial area, the ventromedial nucleus and the lateral hypothalamic area in zebra finch (*Taeniopygia guttata*), areas involved in hypothalamic control over feeding behavior (145, 146). The findings are similar in mammals, where the presence of 26RFa/QRFP has been shown in regions that are important for regulation of food intake and energy homeostasis. In rodents, high levels of 26RFa/QRFP are found in dorsolateral and mediobasal hypothalamic areas, and in humans 26RFa/QRFP-cells are found in the paraventricular and ventromedial nuclei of the hypothalamus (140, 144, 147). It has been found that injections of 26RFa/QRFP have an orexigenic effect (increased food intake) in rodents (148). 26RFa/QRFP has also been found to affect aldosterone secretion, insulin secretion, adipogenesis, bone formation, nociceptive transmission, blood pressure and, as in fish, to stimulate pituitary hormone secretion in mammals (4).

### 26RFa/QRFP in development

The developmental expression of 26RFa/QRFP and GPR103 has only been studied in human adrenal gland and rat adrenals (149). Both the receptor and ligand were present from early stages of adrenal development, mostly expressed in the adrenal cortex, but also in the medulla in human fetus. Some clues for the potential role of 26RFa/QRFP in development are also obtained from knockout studies of the receptor for 26RFa/QRFP in mice (150). Homozygous mice was viable, but they suffered from osteopenia (reduced bone density), and the investigations suggested that the bone formation was arrested at an early stage. It remains unclear if this effect is due to lack of hypothalamic signaling of 26RFa/QRFP, because mRNA of the receptor is found in bone and in osteoblast cell lines in addition to the hypothalamic expression (150). The knockout mice seemed to behave normally and were fertile. However, studies of the brain and the behavior of the animals was not included in the paper, thus it is unknown if knockout of the 26RFa/QRFP receptor can affect specific behaviors.

## Conclusion

The RFa form a complex family with many different members acting in various physiological processes, with one peptide seemingly having several functions in the same animal in some cases. Common to all the peptides seems to be that they could have a role in appetite regulation, pain modulation, and reproduction. With the newest member of the RFa family found only a little over 10 years ago, the field of RFa is relatively new and requires much more research. Especially, how these peptides can influence development is poorly understood. However, the few studies of RFa in developing vertebrates show interesting results that may indicate that many of the RFa could have a separate role in development. Interestingly, it seems that all RFa are expressed early in development in many different groups of vertebrates. However, most of our knowledge of RFa comes from *in situ* hybridization and immunohistochemistry experiments. Very few functional studies have been conducted, so it is difficult to assess the role of this early expression. One knockdown study on medaka performed in our laboratory shows that *kiss1*, *gpr54-1*, and *gpr54-2* play vital roles in early development in this fish species, and that these genes are probably important for proper brain development. It will be very interesting to see if loss or gain of function could reveal novel functions of the other RFa.

The cellular pathways of RFa are poorly understood, and more research is required to find out how RFa can act on developmental processes. However, some RFa (26RFa and Kiss) have been shown to affect migration in cancer cells (114, 151). RFa may also be important for the proper migration of neurons in the developing brain. However, more research is needed to clarify the role of RFa in neuronal migration. Another interesting aspect of RFa is the fact that many of them can affect apoptosis and cell-cycle progression, possibly through affecting opioid receptors (152). PrRP is also found to influence human lymphocyte proliferation (153). In invertebrates, FMRFa has been found to inhibit apoptosis in a snail, indicating that the link between RFa and apoptosis is an evolutionary conserved mechanism (154). Interestingly, NPFF gene expression in mammals is regulated by transcription factors also involved in cell-cycle regulation and apoptosis (155).

The field of RFa in vertebrates is exiting and rapidly expanding. The few developmental studies that have been done show promising and important results. Taken together, these studies indicate that RFa may have a role in development of the nervous system not yet identified. More research is needed, especially functional studies that can give insight into the role these peptides play in development.

## Conflict of Interest Statement

The authors declare that the research was conducted in the absence of any commercial or financial relationships that could be construed as a potential conflict of interest.
